# How Sleep Quality Relates to Bodily and Oral Symptoms: An Analysis from Japanese National Statistics

**DOI:** 10.3390/healthcare10112298

**Published:** 2022-11-17

**Authors:** Yasuno Yokoi, Akira Komatsuzaki

**Affiliations:** 1 Oral Environment and Community Dental Health, The Nippon Dental University Graduate School of Life Dentistry at Niigata, Niigata 951-8151, Japan; 2 Department of Preventive and Community Dentistry, School of Life Dentistry at Niigata, The Nippon Dental University, Niigata 951-8151, Japan; 3 Department of Dental Hygiene, College at Niigata, The Nippon Dental University, Niigata 951-8580, Japan

**Keywords:** sleep quality, symptom, disease, regular hospital visit, quality of life

## Abstract

Background: Sleep is one of the most important health-related factors. This cross-sectional study focused on sleep quality relates to systemic symptoms, including dental symptoms. Methods: Resource data were compiled from 7995 men and women aged 30 to 69 years, which is the core of the Japanese working population. The subjects were divided into four groups based on their answers to two questions, one on sleep time and one on sleep sufficiency, and groups were compared with other items in the questionnaire by means of a contingency table analysis (χ^2^ test). Results: Relationships were found between the sleep groups and basic attributes, the presence of subjective symptoms, and the presence of hospital visits. The items with significant relationships included 14 symptoms, such as lower back pain (*p* < 0.01) and four diseases, including high blood pressure (*p* < 0.01). A multinomial logistic regression was conducted with the sleep groups as objective variables. In the poor sleep group, significant odds ratios were found for four items, including hours of work (odds ratio: 2.53) and feeling listless (2.01). Conclusions: The results allowed multiple symptoms and diseases related to sleep quality to be identified, and different trends in the response rates of the groups were found. These results suggest that the useful classification of sleep quality groups according to health problems contributes to understanding the effects of different symptoms.

## 1. Introduction

Ensuring rest and recuperation through sleep quality is an important factor in improving quality of life [1]. Sleep helps restore vital functions, and sleep disturbance has an impact on general and oral health [2].

Sleep disorders are recognized as a major problem at the international level, and in 2013, the International Classification of Sleep Disorders (ICSD) was put forward by the American Academy of Sleep Medicine [3]. In addition, the International Classification of Diseases 11th Revision (ICD-11) of the World Health Organization has a separate chapter for sleep–wake disorders [4], indicating that sleep disorders are now recognized as diseases.

In addition, there have been numerous reports of an association between sleep quality and lifestyle [5,6,7], and in countries where there is a need to focus on measures for lifestyle-related diseases, it is important to clarify the risk factors that are part of the backdrop to symptoms and diseases. Based on this evidence, we also believe that sleep has a significant impact on health.

In Japan, a major research study investigating the relationship between sleep time and mortality commenced in 1988, and it was reported in 2004 that sleep deprivation was related to increased risk of mortality [8]. It was also reported that approximately 2.5–15% of the population experience drowsiness during the day as a result of sleep deprivation, and 23% are aware of their own sleep deprivation [9]. However, the impact of sleep quality problems is understudied in the Japanese population.

The purpose of the present study is to evaluate the association between sleep quality and various systemic symptoms. We hypothesize that groups with worse sleep quality are characterized by manifestations of symptoms and diseases.

Considering this background, we continue to conduct descriptive epidemiological studies on the relationships between stress, lifestyle habits, and health [10]. Our previous studies [10,11] have suggested that poor sleep quality is detrimental to health. Since FY2013, questions relating to sleep have been added to the Comprehensive Survey of Living Conditions, a major and important set of health-related national statistics. This allows comparisons of sleep risk factors predicted to be related to sleep quality, such as social attributes, lifestyle habits, and medical and dental health statuses. With permission from the Ministry of Health, Labor, and Welfare, we use these anonymized data to clarify how basic lifestyle habits, such as smoking and alcohol consumption, relate to awareness of bodily and oral symptoms and regular hospital visits [11,12,13].

In the field of dentistry, dental diseases that have been reported to have a relationship with sleep include sleep apnea [14] and functional disorders, including bruxism [15]. Treatment for sleep apnea is also performed in dentistry. Problems with sleep are speculated to affect general and oral dysfunction. Sleep quality affects the health of the whole body, and its effects are not only limited to oral health [16].

From a societal point of view, concerns have been raised over the negative impact of working-age individuals’ sleep deprivation [17], and there is growing awareness that sleep disorders are harmful to the health of an individual over a long time period, as well as being responsible for economic losses through undermining productivity.

In this study, we analyze the most recent national statistical data in order to identify factors relating to sleep quality that are associated with symptoms and diseases among the middle-aged and elderly, which are working-age groups. From this study, identifying health risks of sleep quality can increase understanding of disease prevention, with a greater focus on occupational health.

## 2. Materials and Methods

### 2.1. Subjects and Study Design

**Figure 1:**
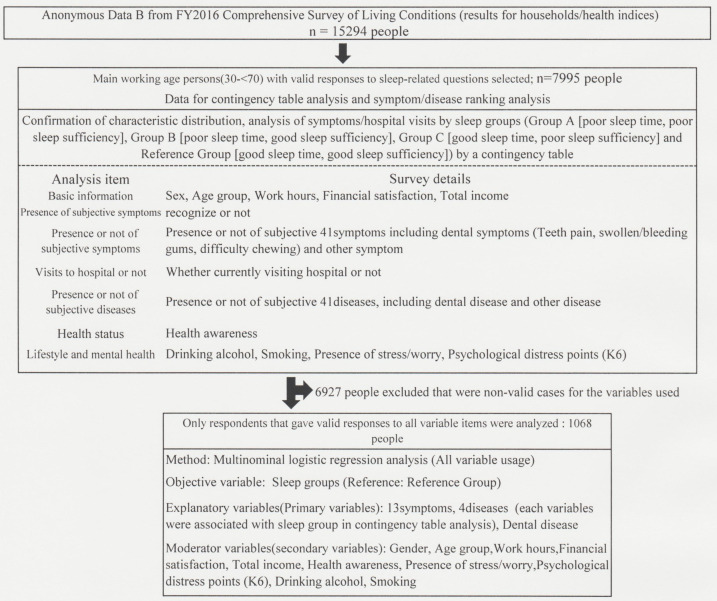
Flowchart of the data analysis in the present study.

The subjects (anonymized individual data) were 7995 persons (3883 men and 4112 women) aged from 30 to 69 years in FY2016. The sample file was drawn so that the distribution was not biased by sex or age strata, and all data were in categorical data format. The subjects were extracted for analysis from the household survey (survey of sex, age, household structure, household economic consciousness, etc.) and the health survey (survey of symptoms, hospital visits, lifestyle habits, health awareness, etc.) in accordance with the stepwise procedure shown in Figure 1. This was a cross-sectional study.

### 2.2. Subject Selection and Classification into Sleep Groups

All subjects were divided as follows into four groups for comparison based on their responses to the question on sleep time (<6 h, poor; ≥6 h, good) and the question on sleep sufficiency (hardly sufficient or not sufficient at all, poor; sufficient or more or less sufficient, good): Group A (poor sleep time, poor sleep sufficiency), Group B (poor sleep time, good sleep sufficiency), Group C (good sleep time, poor sleep sufficiency), and the Reference Group (good sleep time, good sleep sufficiency). The 7995 people grouped into these four groups were extracted and analyzed.

### 2.3. Comparison between Sleep Groups of Response Rate for Each Survey Item

The response rate for each survey item was compared among sleep groups by means of a contingency table (χ^2^ test, Cochran–Armitage trend test) to check for relationships with each sleep group. A residual analysis of items for which significant differences were found in the χ^2^ test was also performed in order to understand trends in distribution at the category level.

### 2.4. Investigation of Degree of Effect on Sleep Status by Multinomial Logistic Regression

Survey items for which relationships with sleep groups were confirmed in the contingency table were examined by means of a multinomial logistic regression. A multinomial model was drawn up with these survey items as explanatory variables (sleeplessness was excluded because it overlapped with the objective variable) and sleep group as the objective variable, and all 1068 valid cases were analyzed. Adjusted odds ratios for each variable were obtained for Groups A, B, and C with respect to the Reference Group.

### 2.5. Comparison between Sleep Group Rankings of Response Rates for Symptoms and Diseases Requiring Hospital Visits

Spearman’s rank correlation matrix and Friedman’s mean rank difference test were used for comparison among sleep groups of the ranking of response rates for symptoms and for diseases requiring hospital visits.

### 2.6. Statistical Analysis

This study used Office Excel (Microsoft, WA, USA) for basic data aggregation. BellCurve for Excel (BellCurve, Tokyo) was used for the χ^2^ test; the Cochran–Armitage trend test, a rank correlation analysis, and SPSS Statistics Ver. 26 (IBM Japan, Tokyo) were used for multinomial logistic regression. The level of significance was set at *p* < 0.05 for all tests.

### 2.7. Ethical Considerations

The data analyzed in the present study were the results of a national survey carried out in line with the Japanese regulations on surveys and were processed for anonymization by the Ministry of Health, Labor, and Welfare. Permission to conduct the study was obtained in accordance with the provisions of Article 36 of the Japanese Statistics Act (Government Statistics 0413 No. 3). All subjects gave their informed consent for inclusion before they participated in the study. The study was conducted in accordance with the Declaration of Helsinki, and the protocol was approved by the Ethics Committee of the School of Life Dentistry at Niigata, the Nippon Dental University (approval No. ECNG-R-398).

## 3. Results

### 3.1. Classification by Sleep Quality

The sleep groups were as follows: Group A, 1474 persons (18.4%); Group B, 1818 (22.7%); Group C, 567 (7.1%), and Reference Group, 4136 (51.7%). The Reference Group (good sleep time, good sleep sufficiency) accounted for the majority of the subjects (Table 1).

### 3.2. Comparison of Sleep Groups by Contingency Table

The survey items by sleep group and the contingency table analyses are shown in Table 2, Table 3, Table 4 and Table 5. In the comparison of response rates for items, such as basic attributes, health awareness, and lifestyle habits (Table 2), the χ^2^ test showed significant differences for sex and all the other items, with a total of 10 items. In the trend test, significant differences were found for sex, age group, hours of work, presence of stress or worry, and alcohol consumption. Contrary to our expectations, in Groups A and B, there were greater response rates for not drinking alcohol.

Those who worked long hours (223 persons, 31.1%) gave low self-evaluations of health (347 persons, 37.0%) and had high K6 scores (294 persons, 40.1%), tending to represent a higher ratio in Group A.

A comparison of the ratio of respondents from each sleep group for each symptom is shown in Table 3. In all cases, the Reference Group accounted for the greatest proportion of respondents, although a slight tendency was found for a greater proportion of people with symptoms in Group A. The results of the trend test showed significant differences for all the symptoms (*p* < 0.01 or *p* < 0.05).

High-ratio poor sleep items (total ratio of Groups A and B above 40%) showed significant differences, including musculoskeletal symptoms such as lower back pain and stiff shoulders. The 14 survey items that showed significant differences in the χ^2^ test also showed significant differences in the trend test. 

The proportions of subjects with diseases that were named in responses are compared between groups in Table 4. Significant differences were found for hospital visits and four diseases (high blood pressure, lumbago, stiff shoulders, and depression or other mental illness). While many persons responded with dyslipidemia and diabetes, there were no significant differences in these categories. High-ratio poor sleep items showed significant differences, including items related to fatigue-susceptible diseases. The category of other diseases also tended to have a high percentage of respondents in Group A (22.4%). On the other hand, depression was higher in Group C (14.8%).

Table 5 illustrates the characteristics of dental symptoms and disease by sleep quality group. Compared to systemic symptoms, dental symptoms were characterized by a higher proportion of Group A respondents. Toothaches and mastication disorders were higher in Group A, both of which were over 30%. However, no significant differences were observed for dental symptoms or hospital visits (χ^2^ test).

### 3.3. Comparison of Symptom and Disease Response Rates

A comparison of the response rates for the five top-ranked symptoms and the five top-ranked injuries or diseases are shown in Table 6. The results for symptoms (frequency of persons reporting the symptom) show that musculoskeletal symptoms, such as lower back pain (966 persons, 37.6%) and stiff shoulders (940 persons, 36.6%), were common. The results for hospital visits for injury or disease show that high blood pressure (944 persons, 28.2%) was the most frequent symptom, after which responses greater than 10% were seen for dyslipidemia (14.5%), dental disease (13.9%), lumbago (11.7%), and diabetes (11.7%). A comparison between the sleep groups in the rank orders of symptoms and of injury or disease by means of rank matrices showed rank correlation among all the groups.

The results of the mean rank difference test (shown in footnote to Table 6) showed significant differences between Group A and Groups B and C (*p* < 0.01), as well as between Group B and the Reference Group (*p* < 0.01), for symptoms and between all the groups except between Groups A and B (*p* < 0.01) for injury or disease.

### 3.4. Results of Multinomial Logistic Regression

In the multinomial logistic regression (Table 7), significant odds ratios were found in Group A for four items: hours of work (odds ratio: 2.53), feeling listless (2.02), health awareness (1.82), and presence of worry or stress (1.92); however, the odds ratio for dental disease (1.51) was not significant (*p* = 0.07). No items in Group B showed significant odds ratios. In Group C, significant odds ratios were found for four items, including presence of worry or stress (4.53) and abdominal or stomach pain (2.51). The coefficient of determination (Cox–Snell) of the analytical model was 0.19, and the likelihood ratio test was significant (*p* < 0.001).

## 4. Discussion

In the present study, about half of the subjects were judged to have problems with either sleep time or sleep sufficiency or both, suggesting that there is a considerable need to examine the effects of sleep on health.

We detected the relationships between sleep groups and demographic characteristics (sex, age, hours of work, total income, health status, and mental condition), the presence of subjective symptoms, and the presence of disease. The symptoms with significant relationships were musculoskeletal symptoms, and the diseases requiring hospital visits with significant relationships were high blood pressure, lumbago, stiff shoulders, and depression or other mental illness. The results suggest that the effects of poor sleep quality occurred both acutely and chronically and were particularly associated with fatigue. Furthermore, these results proved our hypothesis.

In this epidemiological study investigating factors that influence sleep quality, the appearance of symptoms was examined based on the idea that the stage at which people become aware of physical effects as symptoms is critical for considering prevention measures. This is similar to the methods of previous studies [10,11,12,13].

In light of this situation, the Health Japan 21 national health strategy, which commenced in 2000 with the aim of promoting good health in Japan, set targets with regard to sleep. These included the target of reducing the number of persons not obtaining sufficient rest and recuperation through sleep [18].

The results of this study confirmed the possibility that factors relating to symptoms and regular hospital visits may be background factors in the determination of sleep quality, suggesting the need to conduct a broad-based descriptive epidemiological analysis. The results also suggest that examining different combinations of the results for the two sleep-related items in the Comprehensive Survey of Living Conditions allowed an understanding of the effects of differences in the self-evaluation of sleep quality.

The Comprehensive Survey of Living Conditions is the largest Japanese national statistic of living and public health. The survey method is a large-scale sample conducted every 3 years and a small-scale sample every year. The year 2016 was a large-scale sample year, and the purpose of that survey was to gather information on the income, living and health conditions, and expenditures of households in Japan in order to inform program planning. Data were collected through self-completed questionnaires [19]. Recent surveys conducted after 2016 have also included questions about sleep quality.

The results of these data also pointed out problems faced by workers who work long shifts. As a background to fatigue resulting from employment, Dement et al. pointed to the relationship between working patterns, such as those related to the working of night shifts or labor in varying types of industry, and the degradation of sleep habits [17]. There are also concerns in Japan that this may be a major factor causing reduced productivity [20]. The results of the present study show that hours of work had the greatest effect on sleep and that symptoms of fatigue were related to sleep, which may need to be considered in future occupational health measures.

Inadequate sleep time or sleep disorders have been cited as risk factors for lifestyle diseases [21,22,23,24], and it is likely that the physical effects of sleeplessness and overwork disrupt the balance of the sympathetic nervous system [25], leading to the onset of a variety of symptoms. A large number of prior studies comparing sleep status against health indices have mainly surveyed autonomic function and impairment [26,27,28,29,30], and it appears that the effects of sleep are readily reflected in basic physiological responses.

However, Kageyama et al. [27] analyzed how autonomic nervous indices related to sleep time and sleep quality, and they found that, while quality of sleep was related to autonomic indices, sleep time showed no such relationship. This indicated the danger of evaluating the effects of sleep time alone. Methods for the objective evaluation of sleep were subsequently investigated [31], and the Comprehensive Survey of Living Conditions used in the present study contained two questions for surveying sleep.

We also took particular note of diseases that often coexist with sleep deprivation and sleep disorders, including high blood pressure [26], diabetes [32], cardiovascular disease [33], obesity [34], and depression and other mental disorders [35]. The present study yielded the same results as these prior studies, and it appeared likely that improvement in sleep quality leads to improvements in these diseases.

The term “sleep debt” has become commonplace in Japan in recent years, and this is a growing social problem due to its associations with overwork and social networking service addiction [36]. Sleep debt is a term to express the effects of sleep disorders, but an international definition has not yet been decided upon, with a variety of expressions used to describe problems with sleep quality, such as sleep load or sleep tendency [37] and social jetlag [38]. While occupational health measures centered on curbing the number of work hours are being implemented in Japan, the highest odds ratio for hours of work in the present study was 2.53 in Group A, which suggested that further improvement is needed. Nonetheless, it may be conjectured from the present study that the indices for the evaluation of sleep quality were affected by the age or employment status of the subjects, and a multifaceted analysis of sleep quality is needed for future studies.

The odds ratio for the presence of worry or stress was high in Group C, from which it may be conjectured that stress had an effect on the level of sleep sufficiency. Stress is a risk factor for a range of diseases [39], and there are concerns that it has both direct and indirect effects on sleep.

The present analysis did not show any clear association between sleep quality and dental symptoms and dental clinic visits, but the size of the odds ratio for regular dental clinic visits (1.51, *p* = 0.079) suggested the need for further study. Dental diseases reported to have a relationship with sleep include sleep apnea [14]. This was not among the dental symptoms identified in the present study, so analysis was not possible, but some hospitals in Japan have recently opened specialist sleep dentistry departments, and analysis may be expected in the future.

Similarly, the results of this study did not reveal an association between alcohol consumption or smoking and sleep. It is possible that the answers to these negative habit questions are inaccurate, and further investigation is required [40].

The limitations of the study are as follows. First, there was a danger of bias because sleep quality was evaluated subjectively. In addition, as this study was an analysis of the results of a cross-sectional survey, it was difficult to verify causal relationships, and there was a possibility that the results were influenced by unforeseen and unknown confounding factors. A second weakness involved the nature of the questions asked in the national statistics survey, and it was possible that the appropriate survey items were not applied to all the subjects. The sleep quality index assessment was also simple and did not use normal indices (international indicators, such as the PSQI [41]). We would like to overcome these issues by conducting a comprehensive, repeated, multifaceted study to clarify how sleep quality relates to bodily and oral symptoms.

In addition, the study did not examine the health problems of people who sleep for long durations [42] or the effects of sleep medication use [43]. These problems are of particular concern amongst the elderly, and we would like to carry out further studies in the future using other available data to focus on additional factors relating to sleep habits.

## 5. Conclusions

The results of the present study suggested that sleep quality was significantly associated with specific symptoms and diseases. These findings were based on evidence from the Comprehensive Survey of Living Conditions, as well as evaluations of the associations between the sleep quality groups and bodily and oral symptoms. These results suggested the possibility that the recognition of sleep quality may have direct and indirect effects on individuals’ awareness of subjective symptoms and their risk of developing disease.

In conclusion, this study identified different trends in the response rates of symptoms and diseases in subjects with and without high-quality sleep. The classification of subjects into groups based on combinations of responses to two questions regarding sleep allowed an understanding of the effects of differences in the self-evaluation of sleep quality.

Negative health perceptions can affect poor sleep quality; therefore, it is necessary to develop health interventions for symptom management and social support. In particular, it was considered important to strengthen guidance regarding rest in occupational health. Therefore, it is important to conduct further studies on basal sleep needs and how to improve sleep quality in the working population.

## Figures and Tables

**Table 1:** Division into groups by sleep time and sleep sufficiency.

Sleep Groups	Sleep Time: Poor	Sleep Time: Good	Total
	<6 h	≧6 h	
Sleep sufficiency: Poor	Group A	Group C	2041 (25.5)
Hardly sufficient or not sufficient at all	1474 (18.4)	567 (7.1)	
Sleep sufficiency: Good	Group B	Reference (Ref.) Group	
Sufficient or more or less sufficient	1818 (22.7)	4136 (51.7)	
Total	3292 (41.2)	4703 (58.8)	7995 (100.0)

No. of persons (%).

**Table 2:** Comparison of basic attributes, health awareness, and lifestyle habits by sleep group.

Item	Response (Score) *	Group A	Group B	Group C	Ref. Group	Total	*p*: χ^2^ Test	*p*: Trend Test
Sex	Men (1)	638 (16.4)	847 (21.8)	276 (7.1)	2122 (54.7)	3883 (100.0)	<0.001 **	<0.001 **
	Women (0)	836 (20.3)	971 (23.6)	291 (7.1)	2014 (49.0)	4112 (100.0)		
Age group	50s, 60s (1)	702 (15.8)	1038 (23.4)	268 (6.0)	2430 (54.8)	4438 (100.0)	<0.001 **	<0.001 **
	30s, 40s (0)	772 (21.7)	780 (21.9)	299 (8.4)	1706 (48.0)	3557 (100.0)		
Hours of work	≥56 h (1)	223 (31.1)	144 (20.1)	73 (10.2)	278 (38.7)	718 (100.0)	<0.001 **	<0.001 **
	<56 h (0)	960 (18.3)	1214 (23.2)	386 (7.4)	2684 (51.2)	5244 (100.0)		
Financial satisfaction	Poverty (1)	929 (20.2)	1027 (22.3)	364 (7.9)	2281 (49.6)	4601 (100.0)	<0.001 **	<0.001 **
	Normal or affluent (0)	545 (16.1)	791 (23.3)	203 (6.0)	1855 (54.7)	3394 (100.0)		
Total income	<JPY 4 million (1)	388 (16.4)	544 (22.9)	156 (6.6)	1283 (54.1)	2371 (100.0)	<0.001 **	0.002 **
	≥JPY 4 million (0)	1086 (19.3)	1274 (22.7)	411 (7.3)	2853 (50.7)	5624 (100.0)		
Health awareness	Not good or not very good (1)	347 (37.0)	144 (15.4)	150 (16.0)	296 (31.6)	937 (100.0)	<0.001 **	<0.001 **
	Regular, quite good, or good (0)	1114 (15.9)	1666 (23.8)	408 (5.8)	3817 (54.5)	7005 (100.0)		
Presence of stress or worry	Yes (1)	1098 (27.2)	840 (20.8)	426 (10.6)	1670 (41.4)	4034 (100.0)	<0.001 **	<0.001 **
	No (0)	371 (9.5)	970 (24.7)	137 (3.5)	2448 (62.4)	3926 (100.0)		
Psychological distress points (K6)	≥10 (1)	294 (40.1)	124 (16.9)	104 (14.2)	211 (28.8)	733 (100.0)	<0.001 **	<0.001 **
	0–9 (0)	1153 (16.3)	1650 (23.3)	448 (6.3)	3830 (54.1)	7081 (100.0)		
Drinking alcohol	Yes (1)	407 (15.8)	521 (20.2)	183 (7.1)	1466 (56.9)	2577 (100.0)	<0.001 **	<0.001 **
	No (0)	1056 (19.7)	1279 (23.9)	381 (7.1)	2633 (49.2)	5349 (100.0)		
Smoking	Yes (1)	341 (18.2)	414 (22.1)	168 (9.0)	949 (50.7)	1872 (100.0)	0.003 **	0.806
	No (0)	1121 (18.5)	1389 (22.9)	393 (6.5)	3152 (52.1)	6055 (100.0)		

No. of persons (%); ** *p* < 0.01; * set as variable for multinomial logistic regression.

**Table 3:** Comparison of subjective symptoms by sleep group.

Symptom	Response (Score) *	Group A	Group B	Group C	Ref. Group	Total	*p*: χ^2^ Test	*p*: Trend Test
Subjective symptoms	Yes	678 (26.7)	529 (20.6)	277 (10.8)	1076 (41.9)	2569 (100.0)	<0.001 **	<0.001 **
	No	776 (14.5)	1273 (23.7)	282 (5.3)	3034 (56.6)	5365 (100.0)		
Lower back pain	Yes (1)	311 (32.2)	173 (17.9)	116 (12.0)	366 (37.9)	966 (100.0)	<0.001 **	<0.001 **
	No (0)	376 (23.5)	356 (22.2)	161 (10.0)	710 (44.3)	1603 (100.0)		
Stiff shoulders	Yes (1)	314 (33.4)	175 (18.6)	120 (12.8)	331 (35.2)	940 (100.0)	<0.001 **	<0.001 **
	No (0)	373 (22.9)	354 (21.7)	157 (9.6)	745 (45.7)	1629 (100.0)		
Feeling listless	Yes (1)	187 (43.1)	51 (11.8)	72 (16.6)	124 (28.6)	434 (100.0)	<0.001 **	<0.001 **
	No (0)	500 (23.4)	478 (22.4)	205 (9.6)	952 (44.6)	2135 (100.0)		
Headache	Yes (1)	151 (39.0)	67 (17.3)	44 (11.4)	125 (32.3)	387 (100.0)	<0.001 **	<0.001 **
	No (0)	536 (24.6)	462 (21.2)	233 (10.7)	951 (43.6)	2182 (100.0)		
Numbness of limbs	Yes	93 (30.1)	66 (21.4)	41 (13.3)	109 (35.3)	309 (100.0)	0.064	0.029 *
	No	594 (26.3)	463 (20.5)	236 (10.4)	967 (42.8)	2260 (100.0)		
Blurred vision	Yes	94 (30.9)	66 (21.7)	38 (12.5)	106 (34.9)	304 (100.0)	0.058	0.012 *
	No	593 (26.2)	463 (20.4)	239 (10.6)	970 (42.8)	2265 (100.0)		
Difficulty seeing	Yes	81 (29.2)	67 (24.2)	29 (10.5)	100 (36.1)	277 (100.0)	0.158	0.047 *
	No	606 (26.4)	462 (20.2)	248 (10.8)	976 (42.6)	2292 (100.0)		
Tinnitus	Yes (1)	91 (33.6)	55 (20.3)	30 (11.1)	95 (35.1)	271 (100.0)	0.033 *	0.004 **
	No (0)	596 (25.9)	474 (20.6)	247 (10.8)	981 (42.7)	2298 (100.0)		
Constipation	Yes (1)	88 (34.8)	53 (21.0)	37 (14.6)	75 (29.6)	253 (100.0)	<0.001 **	<0.001 **
	No (0)	599 (25.9)	476 (20.6)	240 (10.4)	1001 (43.2)	2316 (100.0)		
Irritability	Yes (1)	110 (45.1)	38 (15.6)	40 (16.4)	56 (23.0)	244 (100.0)	<0.001 **	<0.001 **
	No (0)	577 (24.8)	491 (21.1)	237 (10.2)	1020 (43.9)	2325 (100.0)		
Swollen, tired feet	Yes (1)	88 (37.0)	44 (18.5)	30 (12.6)	76 (31.9)	238 (100.0)	<0.001 **	<0.001 **
	No (0)	599 (25.7)	485 (20.8)	247 (10.6)	1000 (42.9)	2331 (100.0)		
Sleeplessness	Yes	129 (60.3)	25 (11.7)	35 (16.4)	25 (11.7)	214 (100.0)	<0.001 **	<0.001 **
	No	558 (23.7)	504 (21.4)	242 (10.3)	1051 (44.6)	2355 (100.0)		
Upset stomach or heartburn	Yes (1)	76 (36.5)	35 (16.8)	23 (11.1)	74 (35.6)	208 (100.0)	0.007 **	0.006 **
	No (0)	611 (25.9)	494 (20.9)	254 (10.8)	1002 (42.4)	2361 (100.0)		
Dizziness	Yes (1)	71 (38.8)	30 (16.4)	21 (11.5)	61 (33.3)	183 (100.0)	0.001 **	0.001 **
	No (0)	616 (25.8)	499 (20.9)	256 (10.7)	1015 (42.5)	2386 (100.0)		
Forgetfulness	Yes (1)	61 (33.9)	36 (20.0)	23 (12.8)	60 (33.3)	180 (100.0)	0.049 *	0.011 *
	No (0)	626 (26.2)	493 (20.6)	254 (10.6)	1016 (42.5)	2389 (100.0)		
Cold hands and feet	Yes	53 (32.9)	34 (21.1)	19 (11.8)	55 (34.2)	161 (100.0)	0.165	0.029*
	No	634 (26.3)	495 (20.6)	258 (10.7)	1021 (42.4)	2408 (100.0)		
Palpitations	Yes (1)	55 (34.4)	27 (16.9)	23 (14.4)	55 (34.4)	160 (100.0)	0.026 *	0.035 *
	No (0)	632 (26.2)	502 (20.8)	254 (10.5)	1021 (42.4)	2409 (100.0)		
Abdominal or stomach pain	Yes (1)	60 (37.5)	26 (16.3)	31 (19.4)	43 (26.9)	160 (100.0)	<0.001 **	<0.001 **
	No (0)	627 (26.0)	503 (20.9)	246 (10.2)	1033 (42.9)	2409 (100.0)		

No. of persons (%); ** *p* < 0.01; * *p* < 0.05; * set as variable for multinomial logistic regression.

**Table 4:** Comparison of proportions of different injuries and diseases by sleep group.

Disease	Response (Score) *	Group A	Group B	Group C	Ref. Group	Total	*p*: χ^2^ Test	*p*: Trend Test
Hospital visit	Yes	646 (19.3)	756 (22.6)	264 (7.9)	1680 (50.2)	3346 (100.0)	0.014 *	0.036 *
	No	825 (17.9)	1049 (22.8)	297 (6.4)	2437 (52.9)	4608 (100.0)		
High blood pressure	Yes (1)	140 (14.8)	226 (23.9)	53 (5.6)	525 (55.6)	944 (100.0)	<0.001 **	<0.001 **
	No (0)	506 (21.1)	530 (22.1)	211 (8.8)	1155 (48.1)	2402 (100.0)		
Dyslipidemia	Yes	91 (18.8)	117 (24.2)	28 (5.8)	248 (51.2)	484 (100.0)	0.261	0.829
	No	555 (19.4)	639 (22.3)	236 (8.3)	1432 (50.0)	2862 (100.0)		
Lumbago	Yes (1)	120 (30.6)	72 (18.4)	45 (11.5)	155 (39.5)	392 (100.0)	<0.001 **	<0.001 **
	No (0)	526 (17.8)	684 (23.2)	219 (7.4)	1525 (51.6)	2954 (100.0)		
Diabetes	Yes	67 (17.1)	96 (24.6)	24 (6.1)	204 (52.2)	391 (100.0)	0.273	0.404
	No	579 (19.6)	660 (22.3)	240 (8.1)	1476 (50.0)	2955 (100.0)		
Eye disease	Yes	58 (17.7)	65 (19.9)	23 (7.0)	181 (55.4)	327 (100.0)	0.273	0.077
	No	588 (19.5)	691 (22.9)	241 (8.0)	1499 (49.7)	3019 (100.0)		
Stiff shoulders	Yes (1)	75 (28.7)	52 (19.9)	32 (12.3)	102 (39.1)	261 (100.0)	<0.001 **	<0.001 **
	No (0)	571 (18.5)	704 (22.8)	232 (7.5)	1578 (51.2)	3085 (100.0)		
Other disease	Yes	52 (22.4)	46 (19.8)	23 (9.9)	111 (47.8)	232 (100.0)	0.300	0.410
	No	594 (19.1)	710 (22.8)	241 (7.7)	1569 (50.4)	3114 (100.0)		
Depression or other mental disorder	Yes (1)	44 (24.0)	28 (15.3)	27 (14.8)	84 (45.9)	183 (100.0)	<0.001 **	0.333
	No (0)	602 (19.0)	728 (23.0)	237 (7.5)	1596 (50.5)	3163 (100.0)		
Allergic rhinitis	Yes	43 (23.8)	37 (20.4)	16 (8.8)	85 (47.0)	181 (100.0)	0.395	0.248
	No	603 (19.1)	719 (22.7)	248 (7.8)	1595 (50.4)	3165 (100.0)		

No. of persons (%); ** *p* < 0.01; * *p* < 0.05; * set as variable for multinomial logistic regression.

**Table 5:** Comparison of subjective dental symptoms and hospital visits by sleep group.

Item	Response (Score) *	Group A	Group B	Group C	Ref. Group	Total	*p*: χ^2^ Test	*p*: Trend Test
Dental symptoms **	Yes	113 (31.0)	81 (22.2)	33 (9.0)	138 (37.8)	365 (100.0)	0.100	0.024 *
	No	574 (26.0)	448 (20.3)	244 (11.1)	938 (42.6)	2204 (100.0)		
Tooth pain	Yes	51 (35.9)	27 (19.0)	13 (9.2)	51 (35.9)	142 (100.0)	0.099	0.027 *
	No	636 (26.2)	502 (20.1)	264 (10.9)	1025 (42.2)	2427 (100.0)		
Swollen or bleeding gums	Yes	52 (29.1)	37 (20.7)	19 (10.6)	71 (39.7)	179 (100.0)	0.892	0.445
	No	635 (26.6)	492 (20.6)	258 (10.8)	1005 (42.1)	2390 (100.0)		
Difficulty chewing	Yes	32 (32.3)	22 (22.2)	9 (9.1)	36 (36.4)	99 (100.0)	0.502	0.147
	No	655 (26.5)	507 (20.5)	268 (10.9)	1040 (42.1)	2470 (100.0)		
Hospital visits:	Yes (1)	102 (21.9)	104 (22.3)	34 (7.3)	226 (48.5)	466 (100.0)	0.485	0.225
Dental disease	No (0)	544 (18.9)	652 (22.6)	230 (8.0)	1454 (50.5)	2880 (100.0)		

No. of persons (%); * *p* < 0.05; * set as variable for multinomial logistic regression; ** awareness of three dental symptoms.

**Table 6:** Comparison of symptoms and diseases ranked by response rate.

**Symptoms Rank** **^※^**	**1st**	**2nd**	**3rd**	**4th**	**5th**	**Friedman Test**
**Group A**	Stiff shoulders	Lower back pain	Feeling listless	Headache	Sleeplessness	*p* < 0.01 **
No. of persons (%)	314 (45.7)	311 (45.3)	187 (27.2)	151 (22.0)	129 (18.8)	
**Group B**	Stiff shoulders	Lower back pain	Joint pain in hands and feet	Cough or phlegm	Blocked nose or nasal discharge	
No. of persons (%)	175 (33.1)	173 (32.7)	106 (20.0)	77 (14.6)	73 (13.8)	
**Group C**	Stiff shoulders	Lower back pain	Feeling listless	Joint pain in hands and feet	Cough or phlegm	
No. of persons (%)	120 (43.3)	116 (41.9)	72 (26.0)	47 (17.0)	46 (16.6)	
**Ref. Group**	Lower back pain	Stiff shoulders	Joint pain in hands and feet	Cough or phlegm	Blocked nose or nasal discharge	
No. of persons (%)	366 (34.0)	331 (30.8)	168 (15.6)	141 (13.1)	139 (12.9)	
**Total**	Lower back pain	Stiff shoulders	Joint pain in hands and feet	Feeling listless	Headache	
No. of persons (%)	966 (37.6)	940 (36.6)	442 (17.2)	434 (16.9)	387 (15.1)	
**Disease rank** **^※^**	**1st**	**2nd**	**3rd**	**4th**	**5th**	**Friedman test**
**Group A**	High blood pressure	Lumbago	Dental disease	Dyslipidemia	Stiff shoulders	*p* < 0.01 **
No. of persons (%)	140 (21.7)	120 (18.6)	102 (15.8)	91 (14.1)	75 (11.6)	
**Group B**	High blood pressure	Dyslipidemia	Dental disease	Diabetes	Lumbago	
No. of persons (%)	226 (30.0)	117 (15.5)	104 (13.8)	96 (12.7)	72 (9.5)	
**Group C**	High blood pressure	Lumbago	Dental disease	Stiff shoulders	Dyslipidemia	
No. of persons (%)	53 (20.1)	45 (17.1)	34 (12.9)	32 (12.1)	28 (10.6)	
**Ref. Group**	High blood pressure	Dyslipidemia	Dental disease	Diabetes	Eye disease	
No. of persons (%)	525 (31.3)	248 (14.8)	226 (13.5)	204 (12.1)	181 (10.8)	
**Total**	High blood pressure	Dyslipidemia	Dental disease	Lumbago	Diabetes	
No. of persons (%)	944 (28.2)	484 (14.5)	466 (13.9)	392 (11.7)	391 (11.7)	

※ Comparisons between groups: combinations that were significant in the Scheffe test. Symptom ranks (42 items): Group A vs. B (*p* < 0.01), Group A vs. C (*p* < 0.01), Group B vs. Ref. Group (*p* < 0.01); disease ranks (41 items): Group A vs. C (*p* < 0.01), Group A vs. Ref. Group (*p* < 0.01), Group B vs. C (*p* < 0.01), Group B vs. Ref. Group (*p* < 0.01), Group C vs. Ref. Group (*p* < 0.01); ** *p* < 0.01;

**Table 7:** Results of multinomial logistic regression.

	Explanatory Variable ^※^	Partial Regression Coefficient	Odds Ratio	95% C.I. Lower Limit	95% C.I. Upper Limit	Wald	*p*-Value
Group A	Hours of work	0.93	2.53	1.54	4.16	13.33	<0.001 **
	Feeling listless	0.70	2.02	1.28	3.18	9.12	0.002 **
	Presence of worry or stress	0.65	1.92	1.27	2.89	9.50	0.002 **
	Health awareness	0.60	1.82	1.27	2.63	10.46	0.001 **
	Abdominal or stomach pain	0.53	1.70	0.82	3.53	2.02	0.155
	Palpitations	0.47	1.60	0.77	3.36	1.57	0.210
	Dental disease	0.41	1.51	0.95	2.39	3.08	0.079
Group B	Constipation	0.49	1.63	0.86	3.10	2.21	0.137
	Irritability	0.48	1.62	0.80	3.29	1.79	0.181
	Swollen, tired feet	0.45	1.56	0.84	2.91	1.98	0.159
Group C	Presence of worry or stress	1.51	4.53	2.25	9.15	17.79	<0.001 **
	Abdominal or stomach pain	0.92	2.51	1.12	5.63	5.00	0.025 *
	Health awareness	0.67	1.94	1.24	3.05	8.42	0.003 **
	Irritability	0.56	1.75	0.86	3.58	2.39	0.121
	Feeling listless	0.56	1.75	1.00	3.05	3.87	0.049 *
	Depression or mental disorder	0.54	1.71	0.73	3.97	1.54	0.214
	Palpitations	0.44	1.55	0.62	3.86	0.88	0.349
	Drinking alcohol	0.42	1.53	0.98	2.38	3.46	0.062
	Constipation	0.42	1.52	0.73	3.17	1.23	0.268

※ Shows item with odds ratio > 1.5 (** *p* < 0.01 and * *p* < 0.05).

## Data Availability

Not applicable.

## References

[1] Jakobsson U., Hallberg I.R., Westergren A. (2004). Overall and health related quality of life among the oldest old in pain. Qual. Life Res..

[2] Kredlow M.A., Capozzoli M.C., Hearon B.A., Calkins A.W., Otto M.W. (2015). The effects of physical activity on sleep: A meta-analytic review. J. Behav. Med..

[3] Sateia M.J. (2014). International Classification of Sleep Disorders-third Edition: Highlights and modifications. Chest.

[4] World Health Organization (WHO) International Classification of Diseases 11th Revision. https://icd.who.int/browse11/l-m/en#/http%3a%2f%2fid.who.int%2ficd%2fentity%2f274880002.

[5] Knutson K.L., Van Cauter E., Rathouz P.J., DeLeire T., Lauderdale D.S. (2010). Trends in the Prevalence of Short Sleepers in the USA: 1975–2006. Sleep.

[6] Luckhaupt S.E., Tak S., Calvert G.M. (2010). The Prevalence of Short Sleep Duration by Industry and Occupation in the National Health Interview Survey. Sleep.

[7] Matricciani L., Bin Y.S., Lallukka T., Kronholm E., Dumuid D., Paquet C., Olds T. (2017). Past, present, and future: Trends in sleep duration and implications for public health. Sleep Health.

[8] Tamakoshi A., Ohno Y. (2004). Self-reported sleep duration as a predictor of all-cause mortality: Results from the JACC study, Japan. Sleep.

[9] Liu X., Uchiyama M., Kim K., Okawa M., Shibui K., Kudo Y., Doi Y., Minowa M., Ogihara R. (2000). Sleep loss and daytime sleepiness in the general adult population of Japan. Psychiatry Res..

[10] Komatsuzaki A., Ono S. (2020). Study of the Effects of Recognition of Stress on Symptoms and Regular Hospital Visits: An Analysis from Japanese National Statistics. Healthcare.

[11] Kamoda T., Komatsuzaki A., Ono S., Tanaka S., Yokoi Y. (2020). Association between Drinking Habits and Oral Symptoms: A Cross-Sectional Study Based on Japanese National Statistical Data. Int. J. Dent..

[12] Ono S., Komatsuzaki A., Yokoi Y., Kamoda T. (2020). A Study of the Effects of Smoking on Recognition of Symptoms and Subjective Health. Int. J. Clin. Prev. Dent..

[13] Motoi S., Komatsuzaki A., Ono S., Kikuchi H., Iguchi A., Susuga M., Kamoda T. (2021). Relationship between the Appearance of Symptoms and Hospital Visits in Childhood Based on Japanese Statistical Data. Pediatr. Rep..

[14] Koutsourelakis I., Kontovazainitis G., Lamprou K., Gogou E., Samartzi E., Tzakis M. (2020). The role of sleep endoscopy in oral appliance therapy for obstructive sleep apnea. Auris Nasus Larynx.

[15] Saczuk K., Lapinska B., Wilmont P., Pawlak L., Lukomska-Szymanska M. (2019). Relationship between Sleep Bruxism, Perceived Stress, and Coping Strategies. Int. J. Environ. Res. Public Health.

[16] Magee C.A., Caputi P., Iverson D.C. (2011). Relationships between self-rated health, quality of life and sleep duration in middle aged and elderly Australians. Sleep Med..

[17] Dement W.C., Miles L.E., Garskadon M.A. (1982). White paper on sleep and aging. J. Am. Geriatr. Soc..

[18] National Institute of Health and Nutrition Health Japan 21 (the Second Term). https://www.nibiohn.go.jp/eiken/kenkounippon21/en/kenkounippon21/.

[19] Ministry of Health, Labour and Welfare Japan Comprehensive Survey of Living Conditions. https://www.mhlw.go.jp/english/database/db-hss/cslc-index.html.

[20] Ishibashi Y., Shimamura A. (2020). Association between work productivity and sleep health: A cross-sectional study in Japan. Sleep Health.

[21] Hayashino Y., Fukuhara S., Suzukamo Y., Okamura T., Tanaka T., Ueshima H. (2007). Relation between sleep quality, quality of life, and risk of developing diabetes in healthy worker in Japan: The High-risk and Population Strategy for Occupational Health Promotion (HIPOP-OHP) Study. BMC Public Health.

[22] Remi J., Pollmacher T., Spiegelhalder K., Trenkwalder C., Young P. (2019). Sleep-Related Disorders in Neurology and Psychiatry. Dtsch Arztebl Int..

[23] Kageyama M., Odagiri K., Mizuta I., Yamamoto M., Yamaga K., Hirano T., Onoue K., Uehara A. (2017). Health-related behaviors associated with subjective sleep insufficiency in Japanese workers: A cross-sectional study. J. Occup. Health.

[24] Liu Y., Wheaton A.G., Chapman D.P., Cunningham T.J., Lu H., Croft J.B. (2016). Prevalence of Healthy Sleep Duration among Adults—United States, 2014. MMWR. Morb. Mortal. Wkly. Rep..

[25] Thimgan M.S., Duntley S.P., Shaw P.J. (2011). Changes in Gene Expression with Sleep. J. Clin. Sleep Med..

[26] Amagai Y., Ishikawa S., Gotoh T., Kayaba K., Nakamura Y., Kajii E. (2010). Sleep Duration and Incidence of Cardiovascular Events in a Japanese Population: The Jichi Medical School Cohort Study. J. Epidemiol..

[27] Kageyama T., Nishikido N., Kobayashi T., Kurokawa Y., Kaneko T., Kabuto M. (1998). Self-Reported Sleep Quality, Job Stress, and Daytime Autonomic Activities Assessed in Terms of Short-Term Heart Rate Variability among Male White-Collar Workers. Ind. Health.

[28] Van Dongen H.P., Rogers N.L., Dinges D.F. (2003). Sleep debt: Theoretical and empirical issues. Sleep Biol. Rhythm..

[29] Goto Y., Fujiwara K., Sumi Y., Matsuo M., Kano M., Kadotani H. (2021). Work Habit-Related Sleep Debt; Insights From Factor Identification Analysis of Actigraphy Data. Front. Public Health.

[30] Nakata A. (2011). Effects of long work hours and poor sleep characteristics on workplace injury among full-time male employees of small- and medium-scale businesses. J. Sleep Res..

[31] Hayakawa T., Fujita O., Ishida K., Usami T., Sugiura S., Kayukawa Y., Terashima M., Ohta T., Okada T. (2002). Evaluating mental fatigue in patients with obstructive sleep apnea syndrome by the Maastricht Questionnaire. Psychiatry Clin. Neurosci..

[32] Hayashino Y., Yamazaki S., Nakayama T., Sokejima S., Fukuhara S. (2007). Relationship Between Diabetes Mellitus and Excessive Sleepiness During Driving. Exp. Clin. Endocrinol. Diabetes.

[33] Sejbuk M., Mirończuk-Chodakowska I., Witkowska A.M. (2022). Sleep Quality: A Narrative Review on Nutrition, Stimulants, and Physical Activity as Important Factors. Nutrients.

[34] Liu Y., Carlson S.A., Wheaton A.G., Greenlund K.J., Croft J.B. (2021). Sleep Disorder Symptoms Among Adults in 8 States and the District of Columbia, 2017. Prev. Chronic Dis..

[35] Spiegelhalder K., Regen W., Nanovska S., Baglioni C., Riemann D. (2013). Comorbid Sleep Disorders in Neuropsychiatric Disorders Across the Life Cycle. Curr. Psychiatry Rep..

[36] Kaneita Y., Ohida T., Uchiyama M., Takemura S., Kawahara K., Yokoyama E., Miyake T., Harano S., Suzuki K., Fujita T. (2006). The Relationship between Depression and Sleep Disturbances: A Japanese Nationwide General Population Survey. J. Clin. Psychiatry.

[37] Dement W.C., Vaughan C. (1999). The Promise of Sleep.

[38] Okajima I., Kamada Y., Ito W., Inoue Y. (2021). Sleep Debt and Social Jetlag Associated with Sleepiness, Mood, and Work Performance among Workers in Japan. Int. J. Environ. Res. Public Health.

[39] Karita K., Nakao M., Nishikitani M., Nomura K., Yano E. (2005). Autonomic nervous activity changes in relation to the reporting of subjective symptoms among male workers in an information service company. Int. Arch. Occup. Environ. Health.

[40] Ray L.A., Du H., Grodin E. (2020). Capturing habitualness of drinking and smoking behavior in humans. Drug Alcohol Depend..

[41] Buysse D.J., Reynolds C.F., Monk T.H., Berman S.R., Kupfer D.J. (1989). The Pittsburgh Sleep Quality Index: A new instrument for psychiatric practice and research. Psychiatry Res..

[42] Andreasson A., Axelsson J., Bosch J.A., Balter L.J. (2021). Poor sleep quality is associated with worse self-rated health in long sleep duration but not short sleep duration. Sleep Med..

[43] Masse M., Henry H., Cuvelier E., Pinçon C., Pavy M., Beeuwsaert A., Barthélémy C., Cuny D., Gautier S., Kambia N. (2022). Sleep Medication in Older Adults: Identifying the Need for Support by a Community Pharmacist. Healthcare.

